# Metronidazole caused profound drug‐induced immune thrombocytopenia

**DOI:** 10.1002/ccr3.1334

**Published:** 2017-12-15

**Authors:** Jeffrey Lew, Jeffrey Berenberg

**Affiliations:** ^1^ Hematology Oncology Service Tripler Army Medical Center Hawaii 96859

**Keywords:** Drug‐dependent platelet‐reactive antibodies, drug‐induced immune thrombocytopenia, metronidazole, thrombocytopenia

## Abstract

Metronidazole is commonly prescribed and has not been known to cause drug‐induced immune thrombocytopenia. We have provided clinical and laboratory evidence with DDabs that metronidazole can cause drug‐induced immune thrombocytopenia (DITP). Providers must be aware of metronidazole causing DITP because recognition of thrombocytopenia is critical and cessation of the drug should occur promptly.

## Introduction

Drug‐induced immune thrombocytopenia (DITP) is a process in which a drug mediates platelet destruction via the host immune system. DITP can be life‐threatening if the causative drug is not identified and promptly discontinued. The incidence of DITP is estimated to affect 10 cases per 1 million 1. The typical time course of DITP is a drop in platelet count within 7 days of initiation of a new drug, or 2–3 days if the patient has been previously sensitized to the drug. When the drug is discontinued, platelets usually rise rapidly within 10 days. There are six known mechanisms DITP: hapten‐dependent antibody, drug‐dependent platelet antibody (DDabs), fibrin‐induced thrombocytopenia, drug‐specific antibody, autoantibody induction, and immune complex formation 2. This case documents metronidazole as a cause of drug‐dependent antibody‐mediated platelet destruction. DITP caused by DDabs is rare. Unlike hapten‐dependent antibodies that target covalent bonds between drug–platelet protein complexes, DDabs target hydrophobic interactions. A model described by Bougie postulated that the host's existing pool of immunoglobulins have weak interactions with platelet membrane glycoproteins. When an offending drug is introduced into the circulation, hydrophobic interactions form between drug and platelet glycoproteins. The high affinity of the Fc region of the circulating antibodies stimulates B cells to undergo proliferation, producing more antibodies and greater platelet destruction 3.

## Case Presentation

**Figure 1:**
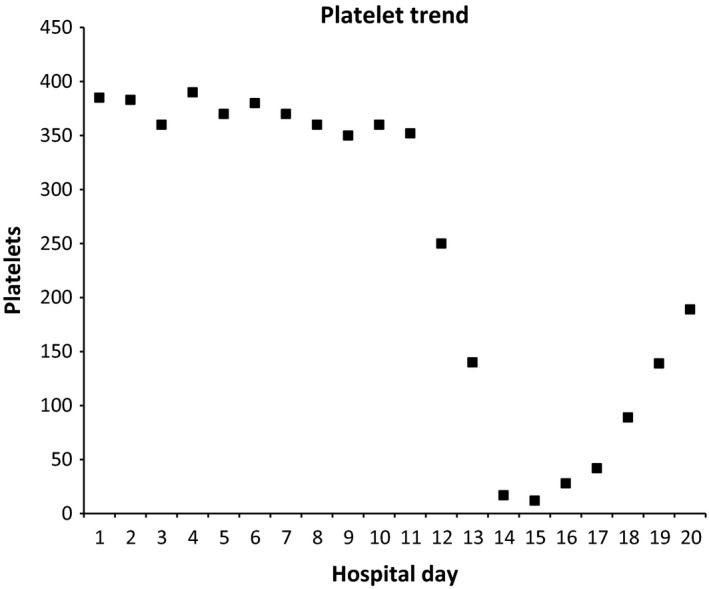
Platelet trend throughout hospital stay.

A 68‐year‐old Caucasian male was hospitalized for a temporal lobe abscess and was treated with vancomycin, metronidazole, and cefepime. After 5 days of antibiotics, the patient underwent a mastoidectomy and drainage of the abscess. He was started on lovenox for DVT prophylaxis on day nine of antibiotics. After 12 days of antibiotic therapy, his platelet count began to decrease, dropping precipitously from 250 × 10^9^/L to 140 × 10^9^/L on day 13 and to 17 × 10^9^/L on day 14 of antibiotics. The mean platelet volume was normal. He was asymptomatic and without the evidence of bleeding. Repeat platelet count using a citrated tube showed a platelet count of 7 × 10^9^/L. Fibrin degradation products, fibrin d‐dimer, fibrinogen, prothrombin time (PT), partial thromboplastin time (PTT), and international normalized ratio (INR) were within normal limits. Peripheral blood smear showed a paucity of platelets, normal platelet size, and no schistocytes. Testing for heparin‐induced thrombocytopenia (HIT) with antiheparin PF4 antibodies was negative (Table 1). DITP was suspected, and antibiotics were changed to daptomycin and moxifloxacin. Over the next 4 days, platelets rose to above 150 × 10^9^/L (Fig. 1). The patient's serum was sent to Blood Center of Wisconsin, where drug DDabs strongly positive to metronidazole and weakly positive to vancomycin were detected by flow cytometry (Table 2).

**Table 1:** Workup for common causes of thrombocytopenia

Test	Result
Platelets in citrated tube	9 × 10^9^/L no platelet clumping
Peripheral blood smear	No schistocytes, paucity of platelets
Fibrin degradation products	<10 (<10) μg/mL
Fibrin D‐dimer	1.14 (0–0.5) μg/mL FEU
Fibrinogen	463 (196–468) mg/dL
PT	14.2 (11.7–14.2) sec
PTT	30 (24–36) sec
INR	1.1
Antiheparin PF4 antibodies	Negative

**Table 2:** Drug‐dependent platelet antibody testing

	IgG result	IgM result
Patient's serum without drug	Negative	Negative
Cefepime	Negative	Negative
Metronidazole	Positive (Strong)	Negative
Pantoprazole	Negative	Negative
Vancomycin	Positive (Weak)	Negative

Drug‐dependent platelet antibody testing by flow cytometry reveals that in the presence of metronidazole IgG antibodies will be bound to the platelet surface and metronidazole forming a complex that will be removed from circulation. Vancomycin has the same interaction; however, it is weakly positive.

## Discussion

Metronidazole is a commonly used antibiotic, and the medical literature has not reported a causal relationship between the drug and DITP. Although metronidazole was reported in the database for BloodCenter of Wisconsin for positive DDabs, there were no accompanying clinical data.

Reese et al. 4 used three systematic methods to identify DITP: case reports, positive drug‐dependent platelet antibodies, and reports cataloged in the United States Food and Drug Administration Adverse Event Reporting System (AERS) database 4. When examining pharmaceuticals in case reports, drugs were classified as having definite, probable, possible, or unlikely association with thrombocytopenia based on four clinical criteria. First, the suspected drug is administered prior to thrombocytopenia and recovery is complete and sustained after cessation of the drug. Second, the suspected drug is the only drug taken before the onset of thrombocytopenia, and other drugs are continued or reintroduced with a sustained normal platelet count. Third, all other potential causes of thrombocytopenia are reasonably excluded, and fourth, re‐exposure to the suspected drug results in recurrent thrombocytopenia. Based on these criteria, this case would represent a “possible” relationship to thrombocytopenia.

The second method was systematic searching through the AERS database with drugs of statistically significant reporting with keywords associated with thrombocytopenia (platelet destruction, idiopathic thrombocytopenic purpura, antiplatelet antibody positive). The third method consisted of reviewing peripheral blood samples submitted to BloodCenter of Wisconsin from 1995 to 2008 for positivity for DDabs.

Of the drugs reviewed by the three systematic methods, 23 were found to both test positive in all three methods, with one drug, amiodarone, positive for the first two methods (Table 3) 5.

**Table 3:** Twenty four implicated drugs for DITP identified by all three methods

Abciximab	Irinotecan	Rifampin
Acetaminophen	Naproxen	Simvastatin
Amiodarone^a^	Oxaliplatin	Sulfisoxazole
Ampicillin	Phenytoin	Tirofiban
Carbamazepine	Piperacillin	Trimethoprim–Sulfamethoxazole
Eptifibatide	Quinidine	Valproic acid
Ethambutol	Quinine	Vancomycin
Haloperidol	Ranitidine	
Ibuprofen		

a One Drug implicated by Methods 1 and 2.

Table adapted from Reese et al. 4.

In this case, two of four clinical criteria were fulfilled: The suspected drug was taken before the onset of thrombocytopenia, and recovery of thrombocytopenia was complete and sustained after the drug was discontinued after all other potential causes of thrombocytopenia were reasonably excluded. However, causality is further supported by IgG antibodies strongly positive in the presence of metronidazole. The combination of clinical criteria and confirmatory antibody testing strengthens the case for metronidazole as a cause of DITP.

There are limitations to establishing the causality of metronidazole and DITP in this case. The patient was never rechallenged with metronidazole after thrombocytopenia occurred; this was considered clinically risky. Additionally, vancomycin is a known cause of DITP, documented in case reports, DDab testing, and in the AERS database. Vancomycin was weakly positive for DDabs and may have contributed to the thrombocytopenia.

## Conclusion

Metronidazole as a cause of DITP has important clinical implications. In patients with thrombocytopenia on metronidazole, DITP should be considered in the differential diagnosis, and the antibiotic should be discontinued. The patient should be flagged as allergic to the drug, to prevent future exposures. The clinical role of DDab testing has not been firmly established, and DITP remains a clinical diagnosis, because there are few centers that offer DDab testing and laboratory protocols for DDab testing are not standardized between these centers 6.

## Authorship

Jeffrey Lew MD, ACP associate, resident physician at Tripler Army Medical Center, and primary author of acquisition of data: involved in the analysis of data and drafting of the manuscript; Jeffrey Berenberg MD, MACP, Staff physician, Department of Hematology‐Oncology Service, Tripler Army Medical Center: involved in critical expertise, analysis, and revision of manuscript.
